# Comprehensive Analysis and Functional Characteristics of Differential Expression of N6-Methyladenosine Methylation Modification in the Whole Transcriptome of Rheumatoid Arthritis

**DOI:** 10.1155/2022/4766992

**Published:** 2022-10-25

**Authors:** Lei Wan, Jian Liu, Chuanbing Huang, Ziheng Zhu, Kun Wang, Guanghan Sun, Lei Zhu, Zhongxiang Hu

**Affiliations:** ^1^The First Affiliated Hospital of Anhui University of Chinese Medicine, Hefei 230038, China; ^2^Key Laboratory of Xin'an Medical Education Ministry, Hefei 230038, China; ^3^College of Traditional Chinese Medicine, Anhui University of Chinese Medicine, Hefei 230012, China; ^4^The First Affiliated Hospital of University of Science and Technology of China, Hefei 230000, China

## Abstract

N6-methyladenosine (m6A) modification is the most prevalent chemical modification in eukaryotic mRNA and is associated with the development of various immune diseases. However, the role of m6A methylation in rheumatoid arthritis (RA) development is unclear. We preliminarily explored the role of m6A methylation-related mRNAs in RA for its clinical application. The discovery of m6A methylation-modifying genes in this study may provide a fresh perspective on the development of drugs for RA treatment. High-throughput sequencing combined with methylated RNA immunoprecipitation (MeRIP-seq) and RNA sequencing were used to assess whole-transcriptome m6A modifications in the synovium of patients with RA. The relationship between m6A-modified target genes and RA inflammation and macrophages was determined. The expression of the m6A-modified significant transcript-enriched inflammatory signaling pathway was assessed through animal experiments. Differentially expressed m6A genes were correlated with macrophage activation involved in immune response, vascular endothelium, MAPK signaling pathway, PI3K − Akt signaling pathway, and other inflammatory processes. Furthermore, combined analysis with m6A-seq and RNA-seq revealed 120 genes with significant changes in both m6A modification and mRNA expression. We selected the top 3 candidate mRNAs that were upregulated and downregulated simultaneously. The expression of phosphatase and tensin homolog deleted on chromosome ten (*PTEN*) *mRNA* and protein in RA patients was lower than that in healthy control (HC). SHC-binding protein 1 (*SHCBP1*) and neurexophilin-3 (*NXPH3*) *mRNA* expressions were increased in RA patients. The expression of M1 macrophages was increased in RA patients. RA markers are such as rheumatoid factor (RF) and peptide containing citrulline (CCP). Further animal experiments showed that the expression of synovial MAPK, PI3K, and Akt1 proteins in the RA model was increased, and the PTEN, p-PTEN protein expression was decreased. PI3K, Akt1, PTEN, and p-PTEN were correlated to RA joint inflammation. This study revealed a unique pattern of differential m6A methylation modifications in RA and concluded that m6A modification is related to the occurrence of RA synovial inflammation.

## 1. Introduction

Rheumatoid arthritis (RA) is the most common autoimmune disease and is characterized by immune cell infiltration, fibroblast-like hyperproliferation of synovial cells, and cartilage destruction [1]. Genetic and epigenetic factors are involved in RA progression [2]. Epigenetic mechanisms play an important role in the occurrence and development of RA [3]. Epigenetic regulation results in heritable changes in gene function without altering DNA sequence, ultimately leading to altered disease phenotypes [4, 5]. RNA methylation plays an important role in maintaining cellular function in RA and refers to methylation modifications that occur at different positions on eukaryotic RNA.

6-Methyladenine (m6A) modification is the most prevalent chemical modification in eukaryotic mRNA [6, 7]. Disturbed m6A modification may lead to abnormal RNA expression [8–10]. m6A modification is catalyzed by specific methyltransferases and is removed by demethylases. Under the activity of the recognition protein read, the modification information is converted into a functional signal [11, 12]. m6A has significance in the regulation of various diseases by interfering with RNA expression [13, 14]. Accumulating evidence suggests that m6A modification is an important regulator of immune response and inflammation regulation [15–17]. m6A is involved in controlling immune recognition and innate and adaptive immune responses [18, 19]. m6A modification plays an important role in the regulation of immunity, viral infection, and auto-inflammatory diseases [16, 20–22]. m6A modification plays an important role in RA disease progression, treatment, and prognosis [3, 23, 24]. m6A methylation modification is also crucial in RA development. The binding protein can recognize the m6A methylation site of mRNA, affecting the abundance and function of mRNAs with m6A modification sites [25, 26]. mRNA expression in RA is also affected [27].

m6A methylation modification is expected to become a new intervention target for the treatment and prevention of RA. However, the research on m6A methylation modification in RA is limited, and further research and exploration are needed. We aimed at investigating the role of m6A modification in RA. In this study, high-throughput sequencing technology was used to screen differentially expressed m6A-modified mRNAs in the RA synovium. The role of m6A modification-related mRNAs in RA was preliminarily explored for its clinical application.

## 2. Materials and Methods

### 2.1. Specimen Source

The RA synovial tissue samples required for RNA methylation coimmunoprecipitation high-throughput sequencing (MeRIP-seq) in this study were all obtained from orthopedic patients. The RA patient samples required for MeRIP-qPCR validation were obtained from the Department of Rheumatology. The specific characteristics of RA patients are described in Table S1. All patients provided their signed informed consent. The research content meets the requirements of ethical review (Ethics No. 2019AH-12).

### 2.2. m6A Immunoprecipitation

Synovial RNA was extracted. Total RNA was extracted with TRIzol (Life Technologies, Waltham, MA, USA). The mRNA was mixed with 2 *μ*g of anti-m6A rabbit polyclonal antibody (Synaptic system, Goettingen, Germany) into a 500-*μ*L IP reaction system and incubated at 4°C for 2 h, followed by incubation with blocked Dynabeads (Thermo, Waltham, MA, USA) for 2 h and purification of the final mRNA.

### 2.3. MeRIP-Seq

For MeRIP-seq, total RNA was isolated using TRIzol reagent. mRNA was further purified using the Dynabeads mRNA DIRECT kit (Thermo Fisher). RNA fragmentation was performed by sonication at 10 ng/ml in 100 ml RNase-free water using Bioruptor Pico (Diagenode) with 30 s on/30 s off for 30 cycles. m6A-IP and library preparation were performed per the reported protocol with some modifications. Briefly, 1 mg sonicated mRNA was mixed with 2.5 mg m6A antibody (Synaptic Systems, 202003) in 1x IP buffer and incubated with head-to-tail mixing at 4°C for 2 h. The mixture was supplemented with 25 mL Protein A magnetic beads (Thermo Fisher) prewashed two times with 1x IP buffer and incubated with head-to-tail mixing at 4C for another 2 h. The beads were then separated and washed with 1x IP buffer three times before eluted with m6A elution buffer for two times. The eluates were combined and purified with RNA Clean and Concentrator (Zymo, Orange, CA). The purified mRNA fragments were then used to construct libraries with TruSeq Stranded mRNA Library Prep Kit (Illumina, San Diego, CA). Sequencing was carried out on Illumina HiSeq 2000 with pair-end 150-bp read length. Reads were aligned to human genome version 38 (GRCh38) with Tophat. The longest isoform was retained if a gene has more than one isoforms. Differential m6A modified peaks between IP and input samples were identified using exomePeak (*p* < 0.01).

### 2.4. RNA High-Throughput Sequencing Analysis (RNA-Seq)

The sequencing quality of reads after de-linking was assessed using the FastQC v0.11.4 software (Babraham Institute, Cambridge, UK). The reference genome was aligned by the Hisat2 v2.1.0 software (University of Texas Southwestern Medical Center, Dallas, TX, USA; https://daehwankimlab.github.io/hisat2/). The gene and transcript levels were calculated using the R software Ballgown v4.0.5 (The University of Auckland, Oakland, New Zealand; https://www.r-project.org/). Differences in the expression at gene and transcript levels were calculated separately. The differentially expressed genes were then screened.

### 2.5. MeRIP-Seq and RNA-Seq Association Analysis

A four-quadrant graph of the “RNA expression” and “m6A modification” (Fold Change ≥1.5, *p* < 0.05) was drawn. Differential m6A modification levels of mRNA and its mRNA expression distribution in the RA synovial tissue were noted. The number of transcripts in each quadrant was then counted.

### 2.6. Real-Time Quantitative PCR

When combined with the MeRIP sequencing results, RMBase v2.0, and SRAMP database information, the methylation modification sites of mRNA were inferred and the relevant primers were designed (Table S5 lists the specific primer sequences). Total RNA was extracted and isolated. The RT kit (TaKaRa, Tokyo, Japan) was used for cDNA synthesis. The synthesized cDNA was subjected to RT-qPCR.

### 2.7. Flow Cytometry

We used heparin sodium anticoagulation tubes to collect 5 ml of peripheral blood, and then took several test tubes. Add antibody PI (10 *μ*l)/CD45-cyanine7 (1 *μ*l)/IgG2b-CY5.5 (1 *μ*l)/IgG1-APC (1 *μ*l) (Biolegend Corp., San Diego, CA, USA) to each tube of 100 *μ*l whole blood as a negative control group. PI(10 *μ*l)/CD45-cyanine7(1 *μ*l)/CD86-CY5.5(1 *μ*l)/CD163-APC(1 *μ*l) (Biolegend Corp., San Diego, CA, USA) was used as the experimental group. Add 1 ml of hemolysin to each test tube. The target cell gate can be set more accurately in the FS/SS graph of the flow cytometer. In CD45/SS flow cytometry, the monocyte gate can be set more precisely. Using a three-color scheme, select the CD45/SS map to set a gate, and detect high expression of CD14 (macrophages) in the blood. As per the antibody manufacturer's instructions, human M1 and M2 macrophages were expressed by adding fluorescently labeled mouse antihuman CD14-FITC, CD86-PE-Cyanine5, and CD163-APC monoclonal antibody reagents (10 *μ*L; Biolegend Corp., San Diego, CA, USA). The mixtures were mixed well and allowed to react in a refrigerator at 4°C for 30 min. The mixtures were centrifuged at 1500 rpm for 5 min to remove the supernatants. The obtained cell deposits were resuspended in PBS for flow cytometry analysis.

### 2.8. Enzyme-Linked Immunosorbent Assay (ELISA)

To estimate the serum levels of inflammatory factors, serum M1 and M2 secreted TNF-*α*, VEGF, and IL-4 (Genemei Biotechnology Corp., Wuhan, China) were diluted as per the ELISA kit instructions. A microplate reader (Leidu Corp., Shenzhen, China) was used to measure the absorbance at 450 nm. The standard substance concentration and the corresponding absorbance value were used to first draw a standard curve and then used to calculate the final serum sample concentrations of TNF-*α*, VEGF, and IL-4 after considering the dilution factor.

### 2.9. Western Blotting

The total synovial proteins were extracted using the protein extraction kit (Beibo Corp., Shanghai, China) and then resolved by electrophoresis. Next, the proteins were transferred from the gel onto the PVDF membrane (Millipore Corp., USA) using the semidry method (Tianneng Corp., China). The PVDF membranes were incubated with respective MAPK, PI3K, Akt1,PTEN,p-MAPK, p-PI3K, p-Akt1, and p-PTEN antibodies (dilution 1 : 1000; Abcam Biotechnology, Cambridge, UK) for 2 h at RT, followed by incubation with the following secondary antibodies for 1 h at RT: antimouse secondary antibody (dilution 1 : 5000. Zhongshan Goldenbridge Biotechnology Corp., Beijing, China) for MAPK, PI3K, Akt1, PTEN, p-MAPK, p-PI3K, p-Akt1, and p-PTEN. The enhanced chemiluminescence method (Thermo) was used to develop the bands, and the images were obtained.

### 2.10. Laboratory Animals

The rats were divided into normal control (NC) group of 10 and adjuvant arthritis (AA) group of 10. Then, 0.1 ml of Freund's complete adjuvant (Sigma, Louis, MO, USA) was intradermally injected into the right hind paw of the AA group rats. An AA arthritis model was established. On the 12th day of injection, 0.05 ml of Freund's complete adjuvant was injected into the tail to boost the immunization. Toe swelling and arthritis index of rats were observed. The rat serum and synovium were collected for experimental research. This experiment was approved by the Animal Experimentation Ethics Committee (AHUCM-rats-2021022).

### 2.11. Statistical Analyses

Statistical processing and analyses were performed using the SPSS 23.0 software (IBM, Armonk, NY, USA) and GraphPad Prism 8.3.0 (GraphPad Software Inc., La Jolla, CA, USA) software. Comparisons between the groups were made using a paired *t*-test. The relationship between the two was analyzed by correlational analyses.

## 3. Results

### 3.1. Transcriptome Distribution Information of Differentially Expressed mRNAs in the RA Synovium

**Figure 1:**
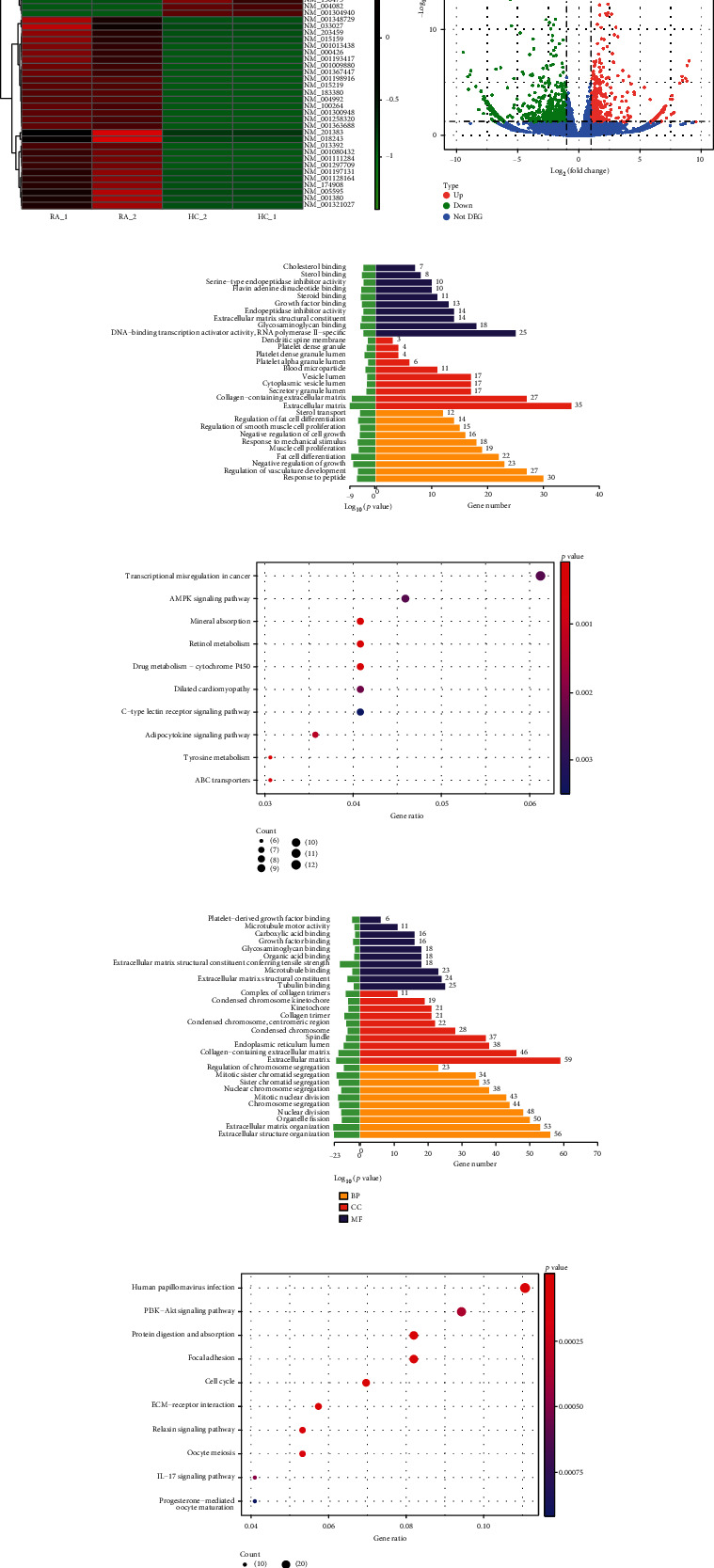
Transcriptome information analysis of differentially expressed mRNA in the RA synovium. (a) Clustering heatmap of differentially expressed genes. (b) Volcano plot of differentially expressed genes. (c) Upregulated mRNAs subjected to GO. (d) KEGG entries for parental genes of the top 10 significantly upregulated transcripts. (e) GO analysis of downregulated genes. (f) KEGG entries for parental genes of the top 10 significantly downregulated transcripts.

We analyzed RA synovial RNA transcriptome information using RNA high-throughput sequencing. Compared with the healthy control (HC) group, the distribution of differentially expressed genes in the RA group showed that the number of upregulated mRNAs was 463. The number of downregulated mRNAs was 603 (Figure 1(a)). Upregulated genes included ANGPTL7, CLSTN2, and MFAP5, whereas downregulated genes included ADAM12, COL5A3, and TOP2A (Figure 1(b)). GO and KEGG analyses were performed. The upregulated genes were mainly enriched in the regulation of vasculature development, muscle cell proliferation, and extracellular matrix. The enriched signaling pathways associated with the differential genes were related to MAPK and Adipocytokine signaling pathways. The downregulated genes were mainly enriched in platelet-derived growth factor binding, collagen-containing extracellular matrix, etc. The enriched signaling pathways were related to PI3K-Akt and IL-17 signaling pathways (Figures 1(c)–1(f)). See Figure S1 and Figure S2 for the KEGG.

### 3.2. Epitranscriptome Distribution of m6A Modification

**Figure 2:**
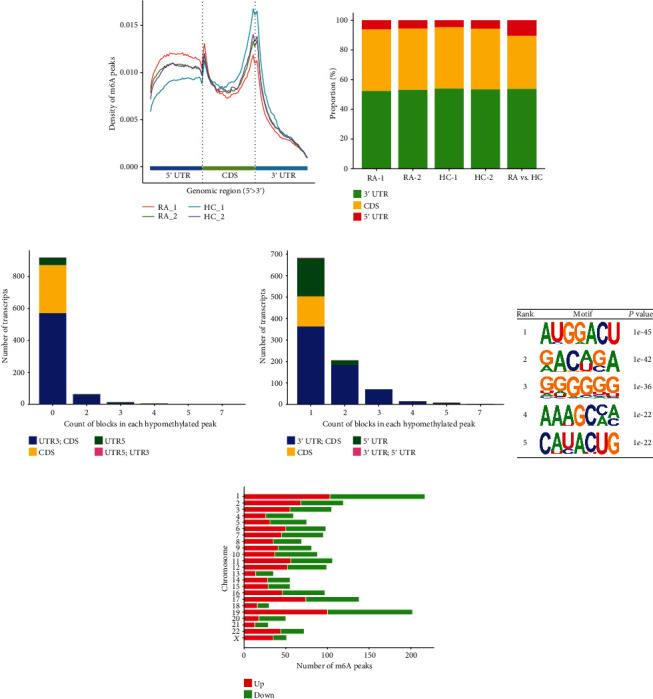
The distribution of m6A-modified epitranscriptomes. (a) Distribution of the peak in the 5'UTR, CDS, and 3'UTR regions. (b) The proportion of differentially methylated peaks in the 5'UTR, CDS, and 3'UTR regions. Counts of peaks whose methylation modifications were (c) downregulated or (d) upregulated in each region. (e) Top 5 m6A-modified motif sequences. (f) Distribution of the genes corresponding to significantly different m6A modification sites in human chromosomes.

Compared with the HC group, 2025 transcripts in the RA synovium had differentially methylated sites (peaks). Among them, 1016 peaks were significantly upregulated and 1009 peaks were significantly downregulated (Fold change >2.0 and *p* < 0.05). In the RA synovial m6A modified transcriptome, the transcripts of 2025 peaks corresponded to 1754 genes. Table S2 enlists the information on the top 10 upregulated and downregulated transcripts. The differential peaks of m6A modification in each sample were mainly distributed in the 3′ untranslated region (3′ UTR) and the adjacent coding region (CDS end) (Figures 2(a), 2(b)). Multiple m6A modification sites were observed on the same transcript (Figures 2(c) and 2(D)). More than half of the methylation modification sites were located in the 3′ UTR region. For transcripts with a peak number of more than 1, the methylation modification sites were mainly located in the CDS region. The consensus sequence for motif enrichment analysis is shown in Figure 2(e). The genes corresponding to differentially methylated transcripts were widely distributed on chromosomes 1, 2, 3, 11, 17, and 19 (number of m6A peaks >100) (Figure 2(f)).

### 3.3. KEGG and GO Analyses of m6A Modified Epitranscriptome

**Figure 3:**
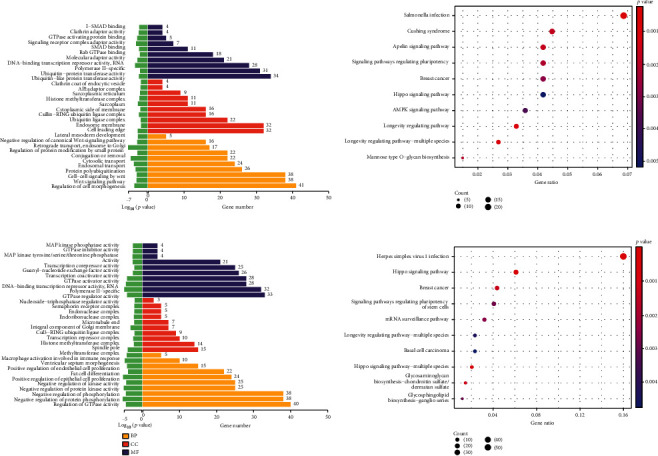
KEGG and GO analysis of m6A-modified epitranscriptome. (a) GO analysis of the upregulated genes corresponding to transcripts with significant m6A modification. (b) KEGG analysis of the upregulated genes corresponding to transcripts with significant m6A modification. (c) GO analysis of the downregulated genes corresponding to transcripts with significant m6A modification. (d) KEGG analysis of transcripts with significant m6A modification corresponding to the downregulated genes.

To further reveal the m6A modification epitranscriptomic information, we screened MeRIP sequencing results. Enriched entries for genes corresponding to the differentially methylated transcripts were obtained. As shown in Figures 3(a) and 3(b), the main enrichment entries of genes corresponding to the upregulated transcripts of methylation modification were cytosolic and endosomal transport, regulation of cell morphogenesis, and cell—cell signaling by wnt. The main enriched items downregulated by methylation modification were macrophage activation involved in immune response, MAP kinase tyrosine/serine/threonine phosphatase activity, etc. KEGG analysis lists the top 10 pathway information of transcripts having significant m6A modification corresponding to the enriched genes (Figures 3(c) and 3(d)). The KEGG signaling pathway analysis showed that the upregulated methylation modifications were mainly enriched in MAPK and the Wnt signaling pathway. The genes corresponding to downregulated transcripts were mainly enriched in the mRNA surveillance pathway and the Hippo signaling pathway. See Figure S3 and Figure S4 for the KEGG heat map.

### 3.4. Correlation Analysis between RNA Sequencing and MeRIP Sequencing

**Figure 4:**
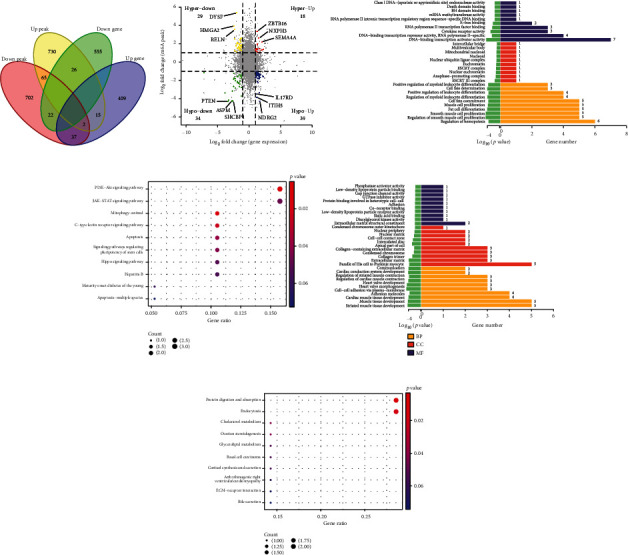
Correlational analyses between RNA sequencing and MeRIP sequencing. (a) Four-quadrant plot of the intersection of differentially expressed genes and the peak. (b) Venn plot of intersection of differentially expressed genes and Peak. (c) GO analysis of the parental genes of significantly upregulated transcripts. (d) KEGG entries for parental genes of the top 10 significantly upregulated transcripts. (e) GO analysis of parental genes of significantly downregulated transcripts. (f) KEGG entries for parental genes of the top 10 significantly downregulated transcripts.

To reveal the regulatory relationship between mRNA m6A modification and its transcriptional level, we performed correlation analysis on MeRIP-seq and RNA-seq data (Figure 4(a)). We found 120 genes with distinct m6A modification peaks and expression changes. As shown in Figure 4(b), 47 transcripts were upregulated by methylation modification and 73 transcripts were downregulated by methylation modification. Among the 47 transcripts upregulated by methylation, 18 transcripts were synchronously upregulated and 29 transcripts reverse downgrades. Among the 73 transcripts downregulated by methylation modification, 34 transcripts were downregulated simultaneously and 39 transcripts reverse upsides. Table S3 enlists the details of the top 5 transcripts in each quadrant.

GO and KEGG analyses of the intersection genes of differentially expressed genes and m6A peak showed that synchronized upregulated differential mRNAs were mainly enriched in mRNA methyltransferase activity, apoptosis, apoptosis—multiple species, cytokine receptor activity, etc. The signaling pathways were the PI3K-Akt signaling pathway, JAK-STAT signaling pathway, etc. (Figures 4(c) and 4(d)). The details of transcripts with differential RNA methylation in the PI3K-AKT signaling pathway are presented in Table S4. Synchronized downregulation of transcript expression and differential mRNA were mainly enriched in muscle tissue development, extracellular matrix, etc. (Figures 4(e)4(f)).

### 3.5. Selection and Clinical Validation of m6A-Modified Target Genes

**Figure 5:**
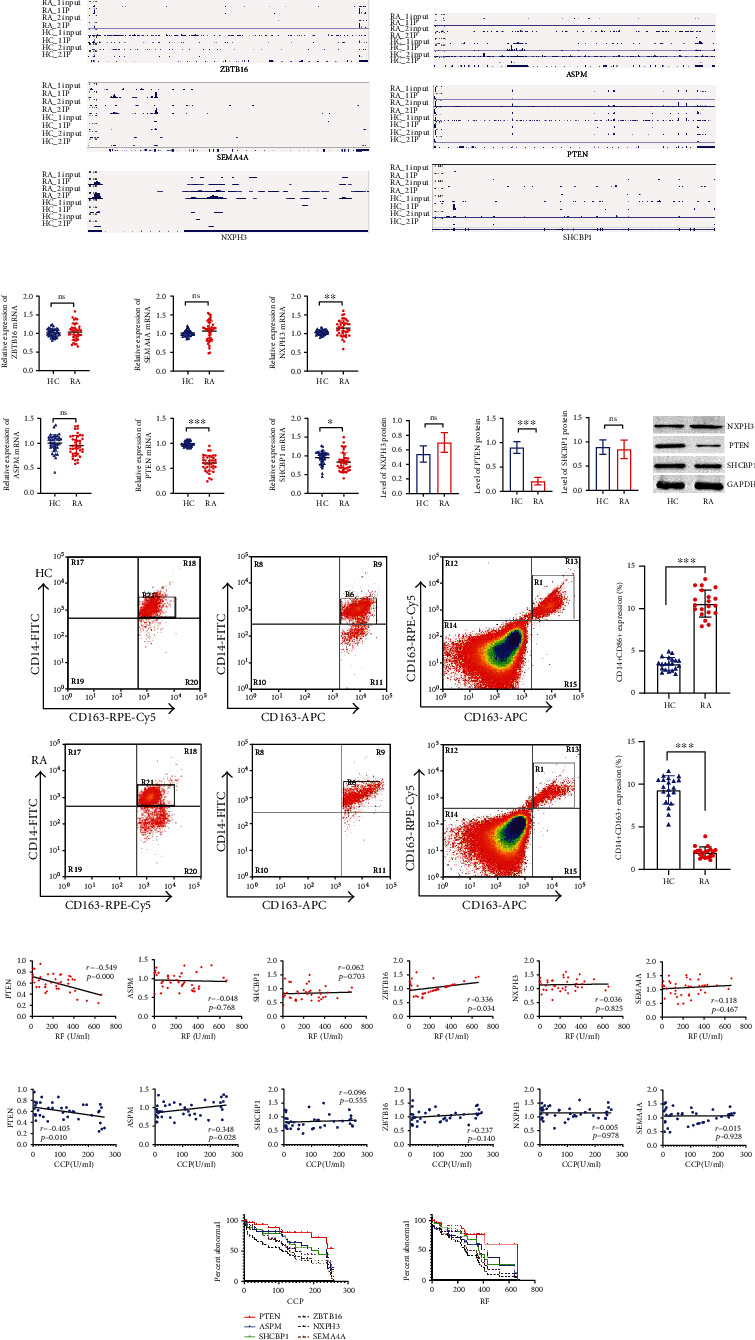
Selection and clinical validation of m6A-modified target genes. (a) Peaks of the upregulated m6A-modified target genes. (b) Peaks of the downregulated m6A-modified target genes. (c) Target genes of m6A modification were detected by RT-PCR. ^∗^*p* < 0.05, ^∗∗^*p* < 0.01, ^∗∗∗^*p* < 0.001 (*n* = 40, paired *t*-test). (d) Protein expression of m6A modification was detected by western blot. ^∗∗∗^*p* < 0.001 (*n* = 6, paired *t*-test). (e) Macrophage marker expression. ^∗∗∗^*p* < 0.001 (*n* = 20, paired *t*-test). (f) Correlational analysis of m6A-modified target genes and RF. (g) Correlational analysis of m6A-modified target genes and CCP. (h) ROC curve analysis of m6A-modified target genes in RA.

After considering the signaling pathway information enriched by MeRIP-seq and combined RNA-sequencing and MeRIP-sequencing association analysis results, we selected the top 3 mRNAs that were upregulated and downregulated at the same time (Table S5). The independent peaks of methylated mRNAs are shown in Figures 5(a) and 5(b). To verify the gene expression of the sequencing results by performing RT-PCR. We collected 40 RA patients and 40 HCs to validate gene expression. The expression of PTEN in the RA group was lower than that in the HC group. The expression of SHCBP1 and NXPH3 in the RA group was higher than that in the HC group. PTEN, SHCBP1, and NXPH3 were expressed in the RA patient when compared with that in healthy person (Figure 5(c)). To further confirm whether there are differences in PTEN, SHCBP1, and NXPH3 proteins. We examined protein expression in RA patients and HC (6/6) by western blot. It was found that PTEN protein in RA patients was lower than that in HC ((Figure 5(d)). Meanwhile, the expression of the M1 macrophage marker (CD86+) was increased, while the expression of the M2 macrophage marker (CD163+) was decreased in patients with RA (Figure 5(e)). The correlation analysis showed that PTEN was negatively correlated with RA disease markers such as RF and CCP. Although ZBTB16 was positively correlated with RF, the expression was not significant in patients with RA. ASPM was negatively correlated with CCP (Figures 5(f), 5(g)). However, ASPM was decreased in patients with RA, which is not in line with clinical practice, ROC curve analysis, and RA disease related to PTEN. Therefore, the most prominently methylated mRNAs in RA patients are PTEN (Figure 5(h)).

### 3.6. The m6A-Modified Inflammatory Signaling Pathway Is Expressed In Vivo

**Figure 6:**
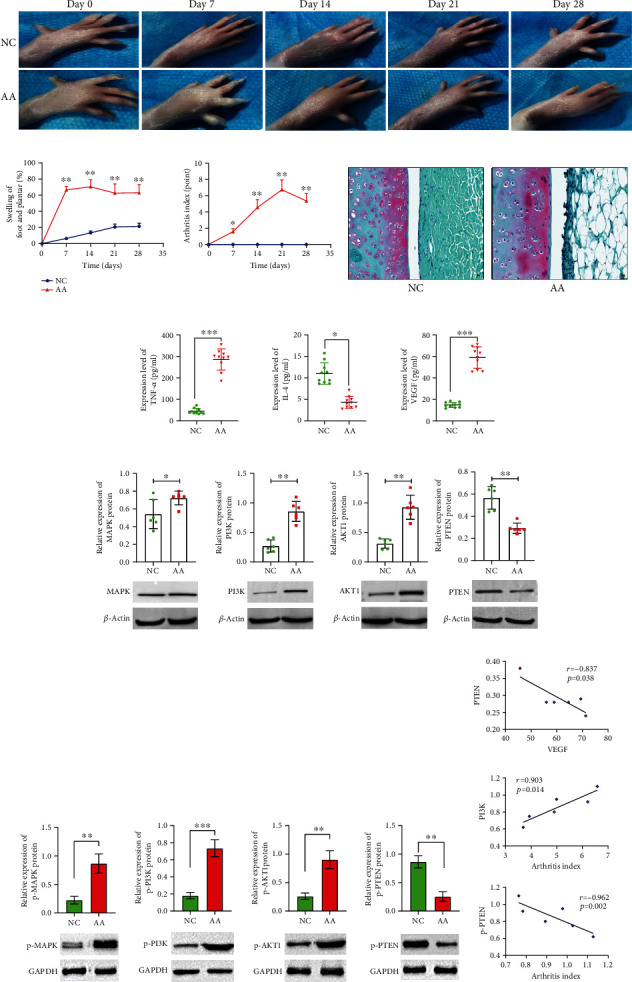
m6A-modified inflammatory signaling pathway is expressed in vivo. (a) Dynamic changes of joint swelling in rats. (b) Comparison of toe swelling and arthritis index. ^∗^*p* < 0.05, ^∗∗^*p* < 0.01 vs NC group (paired *t*-test). (c) Safranin fast-green staining of joints, X400. (d) Comparison of the expression of cytokines secreted by macrophages.^∗^*p* < 0.05, ^∗∗∗^*p* < 0.001 (paired *t*-test). (E) Comparison of the MAPK, PI3K, and AKT inflammatory signaling pathway-related indicators. ^∗^*p* < 0.05, ^∗∗^*p* < 0.01 (paired *t*-test). (f) Comparison of the p-MAPK, p-PI3K, p- AKT1, and p-PTEN.^∗∗^*p* < 0.01, ^∗∗∗^*p* < 0.001 (*n* = 6, paired *t*-test). (g) Correlational analysis of signaling pathway-related indicators and joint inflammation.

We observed increased joint inflammation and articular cartilage destruction in arthritis model rats (Figures 6(a)–6(c)). The expression of cytokines TNF-*α* and VEGF secreted by serum M1 macrophages increased. The expression of IL-4 secreted by M2 macrophages was decreased (Figure 6(d)). Transcripts with significant modification in combination with m6A corresponded to enriched signaling pathways such as MAPK and PI3K-Akt signaling pathways. Further animal experiments found that the expression of synovial MAPK, PI3K, Akt1 proteins, and the phosphorylated protein in the arthritis model was increased, and that of the PTEN protein was decreased (Figures 6(e)–6(f)). Correlation analysis showed that PI3K was positively correlated with arthritis index, PTEN was negatively correlated with VEGF, and p-PTEN was negatively correlated with arthritis index (Figure 6(g)). This indicated that the PI3K-Akt signaling pathway may be involved in joint inflammation.

## 4. Discussion

m6A plays a key molecular role in regulating RNA maturation, localization, translation, and metabolism [6, 24, 26, 28]. m6A methylation modification plays an important role in maintaining stem cell metabolism [29], immune homeostasis [30], tumor immune microenvironment [31], immune suppression and escape [32], and other biological regulations. m6A methylation modification can also affect cell differentiation and proliferation by affecting the expression of transcription factors [33, 34]. m6A methylation is modified by altering RNA folding and structure, which enhances mRNA export and translation [10, 35, 36]. m6A modification regulates cell state by regulating transcription factor expression [37]. m6A methylation also regulates mRNA stability [38]. Therefore, m6A modification may affect disease processes by regulating gene expression and participating in cellular metabolism.

The m6A modification information of RA can be obtained by high-throughput sequencing data analysis. This provides evidence for the pathogenesis and treatment strategies of RA through the regulation of mRNA m6A modification and its transcriptional level in patients with RA. Transcript expression synchronous differential mRNA was mainly enriched in apoptosis, macrophage inflammation, and joint skeletal muscle. These results suggest that m6A methylation modification may be involved in RA cell apoptosis and inflammation, thereby affecting the state of inflammation in the joints and muscles in the body of RA. m6A methylation modification sites are present in different regions of mRNA. It may suggest the transcription- or translation-level information of the corresponding mRNA [14, 39]. Among them, the level of mRNA methylation modification may directly induce an increase or decrease in the protein expression. This shows an RNA-independent translation mechanism independent of the transcription level. The genes in this study showed significant differences in both “m6A modification level” and “gene expression.” For transcripts with the same orientation of “m6A modification level” and “gene expression,” the RA differential peaks were mainly distributed in the CDS region. m6A methylation in the CDS region prevents mRNA degradation. Moreover, analysis of the effect of the mRNA secondary structure on the translation rate is helpful to comprehend the regulatory mechanism of the translation rate [40]. The results of this study showed that m6A methylation modulates the RA process by participating in multiple dimensions such as specific transcription factors and molecules important in synovial fibroblastosis. At the same time, it also provides a theoretical basis for elucidating the mechanism of specific transcripts containing m6A methylation modification.

In the present study, the level of methylation modification of PTEN mRNA was decreased, whereas that of SEMA4A mRNA was increased. This suggests that different m6A methylation modification enzymes and readers may be involved in the regulation of m6A methylation modification of PTEN mRNA and SEMA4A mRNA. The results of this study showed that the parental genes of m6A-modified transcripts were selectively enriched in multiple signaling pathways including PI3K-Akt, MAPK, JAK-STAT, and Wnt signaling pathways. These pathways are interconnected and can be together involved in the processes of RA such as macrophage polarization, inflammatory response, and synovial fibroblast proliferation [41–43]. Interestingly, some genes enriched in PI3K-Akt and MAPK pathways were mostly the repressors of the corresponding pathways including PTEN, ASPM, and SHCBP1. The proliferation and activation of synovial fibroblasts is an important pathological process in RA. Among them, the activation of the PI3K-Akt signaling pathway promotes the proliferation of synovial fibroblasts [44]. PTEN is an antagonist of the PI3K-Akt signaling pathway. As a key negative regulator, PTEN can be overactivated by inhibiting signaling pathways, thereby inhibiting the expression of inflammatory signaling pathways such as PI3K-Akt in RA, as well as an inflammatory response [45]. In the present study, PTEN mRNA expression was decreased in RA patients, while the PTEN expression was decreased in the synovial tissue in the arthritis model, and PI3K and Akt1 protein expressions were increased. Correlation analysis indicated that abnormal expression of signaling pathways was correlated to the level of arthritis inflammation and abnormal secretion of macrophages. This study showed that the low methylation level of PTEN mRNA may provide a new direction in elucidating the mechanism of RA. Therefore, m6A methylation modification site information has specific significance for predicting the fate of mRNA. Further revealing the m6A methylation modification site and the function of the corresponding transcript in RA will be useful.

The discovery of m6A methylation-modifying genes in this study might provide a new perspective on the development of drugs for RA treatment. Notably, there is a gap between m6A modification genes in RA and their clinical application. Therefore, regulating the status of m6A methylation modification may provide a target for the treatment of RA. However, this requires further research. In addition, unraveling the complex regulatory mechanisms associated with m6A methylation modification of drugs may contribute to new strategies for the treatment of RA.

## 5. Contribution and Significance

RA is the most common autoimmune disease. m6A is the most prevalent posttranscriptional modification in eukaryotic mRNA and may affect the disease process by regulating gene expression and by participating in cellular metabolism. The role of m6A methylation modifications in the development of RA is unclear. This study investigated the role of m6A methylation modification in RA using high-throughput sequencing technology for screening differentially expressed m6A-modified mRNAs in the RA synovium. RA synovial m6A modification and mRNA expression were systematically assessed by m6A-seq and RNA-seq. We found that the differentially expressed m6A genes were closely related to inflammatory processes such as macrophage activation, MAPK signaling pathway, PI3K-Akt signaling pathway, and JAK-STAT signaling pathway. To investigate the role of m6A methylation-related mRNAs in RA, the discovery of m6A modification genes in this study will provide a new perspective on the development of drugs for RA treatment. m6A modification has the potential to become a new intervention target for the treatment and prevention of RA. The study findings can be applied to clinical practice. To conclude, this study provides a new strategy for the complex regulatory mechanism of RA and the development of drugs related to m6A modification.

## Data Availability

For more information on how to compose data availability statement, including template examples, please visit: https://www.ncbi.nlm.nih.gov/geo/info/submissionftp.html. The data used to support the findings of this study are available from the corresponding author upon request.

## Supplementary Materials

Supplementary Materials See Table S1‑S5, Figures S1‑S4 in the Supplementary Material for comprehensive analysis. Table S1: basic characteristics of RA patients. Table S2: up- and down-regulated mRNA information of the top 10 differential peaks. Table S3: transcript information of the top 5 in the four-quadrant graph. Table S4: details of 36 transcripts with differential RNA methylation in PI3K-AKT signaling pathway. Table S5: mRNAs with differential m6A modification levels. Figure S1: the KEGG heatmap of upregulated mRNAs distribution information in RA synovium differentially expressed genes. Figure S2: the KEGG heatmap of down-regulated mRNAs distribution information in RA synovium differentially expressed genes. Figure S3: the KEGG heatmap of upregulated peaks in m6A modified apparent transcriptome. Figure S4: the KEGG heatmap down-regulated peaks in m6A modified apparent transcriptome.
