# Supplementary material for: Comprehensive Analysis and Functional Characteristics of Differential Expression of N6-Methyladenosine Methylation Modification in the Whole Transcriptome of Rheumatoid Arthritis
Source: Mediators Inflamm. 2022 Oct 25;2022:4766992. doi: 10.1155/2022/4766992 (PMC9626244; doi:10.1155/2022/4766992)
Supplement: Supplementary Materials — See Table S1‑S5, Figures S1‑S4 in the Supplementary Material for comprehensive analysis. Table S1: basic characteristics of RA patients. Table S2: up- and down-regulated mRNA information of the top 10 differential peaks. Table S3: transcript information of the top 5 in the four-quadrant graph. Table S4: details of 36 transcripts with differential RNA methylation in PI3K-AKT signaling pathway. Table S5: mRNAs with differential m6A modification levels. Figure S1: the KEGG heatmap of upregulated mRNAs distribution information in RA synovium differentially expressed genes. Figure S2: the KEGG heatmap of down-regulated mRNAs distribution information in RA synovium differentially expressed genes. Figure S3: the KEGG heatmap of upregulated peaks in m6A modified apparent transcriptome. Figure S4: the KEGG heatmap down-regulated peaks in m6A modified apparent transcriptome. [file 4766992.f1.zip › Table S1 (1).docx]

| Information | RA(n=40) | | HC(n=40) | |
| --- | --- | --- | --- | --- |
|  | Range（Min,Max） | Mean ±SD | Range（Min,Max） | Mean ±SD |
| Gender |  | |  |  |
| Male(n/%) | 4(10%) | | 5 (12.5%) | |
| Female(n/%) | 36(90%) | | 35 (87.5%) | |
| Age | 65(21,86) | 50.00±16.29 | 58(20,78) | 49.49±15.34 |
| Course of disease | 26.5(0.5,27.0) | 9.65 ±6.38 | NA | NA |
| DAS28 (points) | 8(1,9) | 5.09±1.69 | NA | NA |
| WBC(10^12^/L) | 10.47(2.43,12.90) | 7.44±2.93 | NA | NA |
| Hb（g/L） | 84(78,162) | 120.56±18.64 | NA | NA |
| PLT(10^9^/L) | 329.08(59.19,388.27) | 217.41±88.84 | NA | NA |
| IgG(g/L) | 24.02(2.22,26.24) | 15.40±5.61 | NA | NA |
| IgA(g/L) | 4.60(0.88,5.48) | 2.83±1.23 | NA | NA |
| IgM(g/L) | 2.28(0.46,2.74) | 1.58±.51 | NA | NA |
| C3(g/L) | 1.89(0.68,2.57) | 1.58±.51 | NA | NA |
| C4(g/L) | 0.34 (0.17,0.51) | 0.33±0.07 | NA | NA |
| ESR(mm/h) | 122(6,128) | 54.95±31.23 | NA | NA |
| CRP(mg/L) | 120.71(3.67,124.38) | 55.99±29.99 | NA | NA |
| α1-AGP(mg·d L^-1^) | 199.18(11.40,210.58) | 134.62±36.63 | NA | NA |
| RF(U/ml) | 658.70(3.44,662.14) | 226.47±170.90 | NA | NA |
| CCP(U/ml) | 255.78(2.32,258.10) | 106.31±90.68 | NA | NA |

Table S1 Basic characteristics
