# Supplementary material for: Comprehensive Analysis and Functional Characteristics of Differential Expression of N6-Methyladenosine Methylation Modification in the Whole Transcriptome of Rheumatoid Arthritis
Source: Mediators Inflamm. 2022 Oct 25;2022:4766992. doi: 10.1155/2022/4766992 (PMC9626244; doi:10.1155/2022/4766992)
Supplement: Supplementary Materials — See Table S1‑S5, Figures S1‑S4 in the Supplementary Material for comprehensive analysis. Table S1: basic characteristics of RA patients. Table S2: up- and down-regulated mRNA information of the top 10 differential peaks. Table S3: transcript information of the top 5 in the four-quadrant graph. Table S4: details of 36 transcripts with differential RNA methylation in PI3K-AKT signaling pathway. Table S5: mRNAs with differential m6A modification levels. Figure S1: the KEGG heatmap of upregulated mRNAs distribution information in RA synovium differentially expressed genes. Figure S2: the KEGG heatmap of down-regulated mRNAs distribution information in RA synovium differentially expressed genes. Figure S3: the KEGG heatmap of upregulated peaks in m6A modified apparent transcriptome. Figure S4: the KEGG heatmap down-regulated peaks in m6A modified apparent transcriptome. [file 4766992.f1.zip › Table S2 (1).docx]

Table S2 Up- and down-regulated mRNA information of the top 10 differential peaks

| No. | Gene | Chromosome | Peak Start | Peak End | Peak region | block Count | blockSizes | Log2 Fold Change | P value | type |
| --- | --- | --- | --- | --- | --- | --- | --- | --- | --- | --- |
| 1 | CISH | chr3 | 50607478 | 50607753 | exonic | 1 | 275 | -6.083087539 | 7.42852E-05 | Down |
| 2 | SPC24 | chr19 | 11155735 | 11155785 | exonic | 1 | 50 | -5.30237731 | 0.00581267 | Down |
| 3 | KLF1 | chr19 | 12884622 | 12884797 | UTR3 | 1 | 175 | -5.162566067 | 0.037125852 | Down |
| 4 | FANCF | chr11 | 22624957 | 22625007 | exonic | 1 | 50 | -4.93519357 | 0.002265714 | Down |
| 5 | ZNF823 | chr19 | 11721614 | 11721839 | exonic | 1 | 225 | -4.899616348 | 0.001482132 | Down |
| 6 | CEP170B | chr14 | 104887491 | 104887741 | exonic | 1 | 250 | -4.880764833 | 0.001281412 | Down |
| 7 | PRDM11 | chr11 | 45228015 | 45228165 | exonic | 1 | 150 | -4.650974614 | 0.003074746 | Down |
| 8 | SYNDIG1 | chr20 | 24666285 | 24666335 | UTR3 | 1 | 50 | -4.60588699 | 0.004673451 | Down |
| 9 | ZNF136 | chr19 | 12187761 | 12187811 | exonic | 1 | 50 | -4.605886574 | 0.003346155 | Down |
| 10 | ZNF37A | chr10 | 38124218 | 38124293 | UTR3 | 1 | 75 | -4.605886085 | 0.002562338 | Down |
| 11 | RYR1 | chr19 | 38565545 | 38565670 | exonic | 1 | 125 | 5.811495514 | 0.000202091 | Up |
| 12 | RNF112 | chr17 | 19416002 | 19416277 | exonic | 1 | 275 | 5.478209121 | 0.000473139 | Up |
| 13 | GOLGA6L3 | chr15 | 85244594 | 85244994 | exonic | 1 | 400 | 5.316578779 | 0.000293497 | Up |
| 14 | ZNF784 | chr19 | 55622265 | 55622340 | exonic | 1 | 75 | 5.304481152 | 0.000739751 | Up |
| 15 | DYSF | chr2 | 71453685 | 71453910 | UTR5 | 1 | 225 | 5.279388917 | 0.000515495 | Up |
| 16 | RELT | chr11 | 73391153 | 73392265 | exonic | 2 | 70,55 | 5.13455742 | 0.00180796 | Up |
| 17 | PNMA8B | chr19 | 46493615 | 46493690 | exonic | 1 | 75 | 5.098576732 | 0.001056763 | Up |
| 18 | GPIHBP1 | chr8 | 143215963 | 143216138 | UTR3 | 1 | 175 | 5.064065417 | 0.000939591 | Up |
| 19 | QPRT | chr16 | 29695010 | 29695185 | exonic | 1 | 175 | 5.059477177 | 0.001080964 | Up |
| 20 | RTN4RL1 | chr17 | 2025070 | 2025120 | UTR5 | 1 | 50 | 4.861540048 | 0.003820638 | Up |
