# Supplementary material for: Comprehensive Analysis and Functional Characteristics of Differential Expression of N6-Methyladenosine Methylation Modification in the Whole Transcriptome of Rheumatoid Arthritis
Source: Mediators Inflamm. 2022 Oct 25;2022:4766992. doi: 10.1155/2022/4766992 (PMC9626244; doi:10.1155/2022/4766992)
Supplement: Supplementary Materials — See Table S1‑S5, Figures S1‑S4 in the Supplementary Material for comprehensive analysis. Table S1: basic characteristics of RA patients. Table S2: up- and down-regulated mRNA information of the top 10 differential peaks. Table S3: transcript information of the top 5 in the four-quadrant graph. Table S4: details of 36 transcripts with differential RNA methylation in PI3K-AKT signaling pathway. Table S5: mRNAs with differential m6A modification levels. Figure S1: the KEGG heatmap of upregulated mRNAs distribution information in RA synovium differentially expressed genes. Figure S2: the KEGG heatmap of down-regulated mRNAs distribution information in RA synovium differentially expressed genes. Figure S3: the KEGG heatmap of upregulated peaks in m6A modified apparent transcriptome. Figure S4: the KEGG heatmap down-regulated peaks in m6A modified apparent transcriptome. [file 4766992.f1.zip › Table S3 (1).docx]

Table S3 Transcript information of the top 5 in the four-quadrant graph

| NO. | Gene name | Transcript ID | m6A log2FoldChange | m6A P-value | Gene log2FoldChange | Gene P-value | Change |
| --- | --- | --- | --- | --- | --- | --- | --- |
| 1 | PTEN | NM_000314 | -4.33380 | 0.01155 | -3.26925 | 0.01507 | Hypo-down |
| 2 | ASPM | NM_018136 | -4.29512 | 0.02018 | -2.60292 | 0.00001 | Hypo-down |
| 3 | SHCBP1 | NM_024745 | -4.25813 | 0.00576 | -2.44337 | 0.00052 | Hypo-down |
| 4 | WNT16 | NM_057168 | -4.20070 | 0.03106 | -3.14259 | 0.00122 | Hypo-down |
| 5 | LDLR | NM_000527 | -3.72585 | 0.00016 | -1.09418 | 0.00824 | Hypo-down |
| 6 | NDRG2 | NM_201541 | -4.25170 | 0.00523 | 1.68566 | 0.00001 | Hypo-up |
| 7 | ITIH5 | NM_030569 | -3.81426 | 0.01088 | 1.24603 | 0.02812 | Hypo-up |
| 8 | IL17RD | NM_001318864 | -3.45533 | 0.02727 | 1.09543 | 0.01586 | Hypo-up |
| 9 | TENM1 | NM_001163278 | -3.34850 | 0.03005 | 1.60276 | 0.00094 | Hypo-up |
| 10 | IRF5 | NM_001098629 | -2.25080 | 0.00858 | 1.42239 | 0.00607 | Hypo-up |
| 11 | DYSF | NM_001130981 | 5.27939 | 0.00052 | -1.81384 | 0.00001 | Hyper-dowm |
| 12 | HMGA2 | NM_003483 | 3.85317 | 0.00926 | -2.24754 | 0.00054 | Hyper-dowm |
| 13 | RELN | NM_173054 | 3.47911 | 0.02232 | -2.14897 | 0.00693 | Hyper-dowm |
| 14 | MTHFD1L | NM_015440 | 2.49961 | 0.02839 | -1.09669 | 0.02937 | Hyper-dowm |
| 15 | FLT4 | NM_182925 | 2.43869 | 0.00728 | -2.11196 | 0.00057 | Hyper-dowm |
| 16 | ZBTB16 | NM_006006 | 3.33756 | 0.00001 | 1.27367 | 0.00821 | Hyper-up |
| 17 | NXPH3 | NM_007225 | 2.63251 | 0.00970 | 1.53510 | 0.01225 | Hyper-up |
| 18 | SEMA4A | NM_001193300 | 2.18304 | 0.00006 | 2.03156 | 0.00199 | Hyper-up |
| 19 | AGAP11 | NM_133447 | 1.94288 | 0.00624 | 1.30946 | 0.00504 | Hyper-up |
| 20 | ADIPOQ | NM_004797 | 1.48114 | 0.02165 | 1.61516 | 0.02940 | Hyper-up |
