# Supplementary material for: Comprehensive Analysis and Functional Characteristics of Differential Expression of N6-Methyladenosine Methylation Modification in the Whole Transcriptome of Rheumatoid Arthritis
Source: Mediators Inflamm. 2022 Oct 25;2022:4766992. doi: 10.1155/2022/4766992 (PMC9626244; doi:10.1155/2022/4766992)
Supplement: Supplementary Materials — See Table S1‑S5, Figures S1‑S4 in the Supplementary Material for comprehensive analysis. Table S1: basic characteristics of RA patients. Table S2: up- and down-regulated mRNA information of the top 10 differential peaks. Table S3: transcript information of the top 5 in the four-quadrant graph. Table S4: details of 36 transcripts with differential RNA methylation in PI3K-AKT signaling pathway. Table S5: mRNAs with differential m6A modification levels. Figure S1: the KEGG heatmap of upregulated mRNAs distribution information in RA synovium differentially expressed genes. Figure S2: the KEGG heatmap of down-regulated mRNAs distribution information in RA synovium differentially expressed genes. Figure S3: the KEGG heatmap of upregulated peaks in m6A modified apparent transcriptome. Figure S4: the KEGG heatmap down-regulated peaks in m6A modified apparent transcriptome. [file 4766992.f1.zip › Table S4 (1).docx]

Table S4 Details of 36 transcripts with differential RNA methylation in PI3K-AKT signaling pathway

| No. | Gene name | Transcript ID | Regulation | log2FoldChange  (m6A) | P-value  (m6A) | log2FoldChange  (Gene) | P-value  (Gene) | peak region  (m6A) | Chromosome | Peak Start | Peak End | Block Count |
| --- | --- | --- | --- | --- | --- | --- | --- | --- | --- | --- | --- | --- |
| 1 | AKT1 | NM_001014431 | Up | 1.15831 | 0.00001 | -0.5491 | 0.04503 | exonic | chr14 | 104775158 | 104776761 | 3 |
| 2 | ATF4 | NM_182810 | Up | 1.0906 | 0.00001 | 0.36916 | 0.13693 | UTR5 | chr22 | 39520783 | 39521233 | 1 |
| 3 | CCND3 | NM_001287427 | Up | 1.52637 | 0.01673 | -0.30468 | 0.52076 | UTR5 | chr6 | 42030148 | 42048658 | 1 |
| 4 | CDKN1A | NM_000389 | Up | 1.05905 | 0.00004 | 0.63048 | 0.15458 | exonic | chr6 | 36677849 | 36678925 | 1 |
| 5 | PTEN | NM_000314 | Down | -4.33381 | 0.01155 | -3.26925 | 0.01507 | exonic | Chr10 | 87960893 | 87961118 | 5 |
| 6 | CRTC2 | NM_181715 | Up | 1.21303 | 0.00274 | -0.00035 | 0.99921 | exonic | chr1 | 153955154 | 153958607 | 2 |
| 7 | DDIT4 | NM_019058 | Up | 1.46058 | 0.00009 | -0.90013 | 0.06031 | exonic | chr10 | 72274369 | 72274419 | 1 |
| 8 | EPOR | NM_000121 | Down | -1.50623 | 0.01424 | -0.24485 | 0.74341 | exonic | chr19 | 11378081 | 11378306 | 1 |
| 9 | FGF2 | NM_001361665 | Down | -1.61635 | 0.00003 | -0.23941 | 0.69597 | exonic | chr4 | 122892236 | 122892436 | 1 |
| 10 | FGFR1 | NM_023110 | Up | 1.53893 | 0.00057 | 0.03009 | 0.9184 | exonic | chr8 | 38429755 | 38429930 | 1 |
| 11 | FLT4 | NM_182925 | Up | 2.43869 | 0.00728 | -2.11196 | 0.00057 | exonic | chr5 | 180603005 | 180607763 | 1 |
| 12 | FOXO3 | NM_001455 | Up | 1.08941 | 0.00034 | 0.4455 | 0.07994 | exonic | chr6 | 108559824 | 108561675 | 2 |
| 13 | GHR | NM_000163 | Down | -1.47929 | 0.00601 | 1.08283 | 0.0484 | exonic | chr5 | 42719088 | 42719538 | 1 |
| 14 | GNB5 | NM_016194 | Down | -1.19031 | 0.00452 | 0.22748 | 0.65922 | exonic | chr15 | 52147507 | 52184610 | 5 |
| 15 | GYS1 | NM_002103 | Up | 2.61746 | 0.00001 | 0.39401 | 0.34884 | UTR3 | chr19 | 48968329 | 48968404 | 1 |
| 16 | IFNAR1 | NM_000629 | Down | -2.17722 | 0.00002 | -0.10138 | 0.72582 | UTR3 | chr21 | 33355939 | 33356164 | 1 |
| 17 | IGF1R | NM_000875 | Down | -1.18522 | 0.0001 | 0.27937 | 0.29368 | exonic | chr15 | 98957195 | 98957645 | 1 |
| 18 | IL6R | NM_000565 | Down | -1.26235 | 0.00456 | 1.1582 | 0.0027 | UTR3 | chr1 | 154465510 | 154465860 | 1 |
| 19 | IL7 | NM_001199887 | Up | 2.48051 | 0.03296 | -2.19782 | 0.20335 | exonic | chr8 | 78733671 | 78733771 | 1 |
| 20 | INSR | NM_000208 | Down | -1.04747 | 0.00413 | 0.43165 | 0.12701 | UTR3 | chr19 | 7115889 | 7116314 | 1 |
| 21 | ITGAV | NM_002210 | Up | 1.36385 | 0.00006 | -0.74495 | 0.00079 | UTR5 | chr2 | 186590130 | 186590255 | 1 |
| 22 | ITGB4 | NM_001321123 | Up | 1.15201 | 0.01459 | -0.07788 | 0.76705 | exonic | chr17 | 75721458 | 75724739 | 2 |
| 23 | LAMC2 | NM_005562 | Down | -2.55238 | 0.00427 | -0.73754 | 0.45907 | UTR3 | chr1 | 183243651 | 183243751 | 1 |
| 24 | LPAR5 | NM_020400 | Down | -1.06018 | 0.02466 | 0.55774 | 0.39505 | UTR3 | chr12 | 6619709 | 6620009 | 1 |
| 25 | MCL1 | NM_182763 | Down | -1.34701 | 0.00001 | 1.83178 | 0.00001 | UTR3 | chr1 | 150577000 | 150577325 | 1 |
| 26 | MET | NM_001127500 | Down | -2.23151 | 0.0058 | 0.24475 | 0.51765 | UTR3 | chr7 | 116797957 | 116798207 | 1 |
| 27 | MYC | NM_002467 | Down | -1.60425 | 0.00001 | -0.11343 | 0.80015 | exonic | chr8 | 127740758 | 127741183 | 1 |
| 28 | PGF | NM_001207012 | Down | -1.37049 | 0.00801 | -1.19316 | 0.07066 | UTR5 | chr14 | 74955453 | 74955678 | 1 |
| 29 | PHLPP1 | NM_194449 | Down | -3.65637 | 0.00112 | 0.42421 | 0.36706 | exonic | chr18 | 62979359 | 62979559 | 1 |
| 30 | PHLPP2 | NM_001289003 | Down | -2.81939 | 0.00031 | 0.32293 | 0.52343 | exonic | chr16 | 71648900 | 71649200 | 1 |
| 31 | PIK3CB | NM_006219 | Up | 1.52708 | 0.03917 | 0.0894 | 0.85697 | UTR5 | chr3 | 138796560 | 138834928 | 2 |
| 32 | PIK3CD | NM_001350234 | Down | -2.20516 | 0.02013 | 0.0571 | 0.9106 | exonic | chr1 | 9726949 | 9727149 | 1 |
| 33 | PPP2R5E | NM_006246 | Down | -1.44424 | 0.00061 | 0.02694 | 0.9256 | UTR3 | chr14 | 63375705 | 63375930 | 1 |
| 34 | PTK2 | NM_001352694 | Down | -1.00533 | 0.01492 | 0.14934 | 0.57444 | exonic | chr8 | 140659424 | 140659499 | 1 |
| 35 | RELN | NM_173054 | Up | 3.47911 | 0.02232 | -2.14897 | 0.00693 | exonic | chr7 | 103989295 | 103989470 | 1 |
| 36 | RPS6KB2 | NM_003952 | Up | 1.29196 | 0.00034 | -0.17021 | 0.73303 | exonic | chr11 | 67428493 | 67429223 | 3 |
| 37 | RPTOR | NM_020761 | Down | -1.55452 | 0.00273 | 0.37519 | 0.26741 | UTR3 | chr17 | 80964680 | 80964980 | 1 |
| 38 | SOS2 | NM_006939 | Down | -1.15533 | 0.0053 | 0.18325 | 0.57262 | exonic | chr14 | 50159971 | 50160021 | 1 |
| 39 | SYK | NM_003177 | Down | -2.08739 | 0.0186 | 0.66556 | 0.05318 | UTR3 | chr9 | 90895935 | 90896035 | 1 |
