# Supplementary material for: Comprehensive Analysis and Functional Characteristics of Differential Expression of N6-Methyladenosine Methylation Modification in the Whole Transcriptome of Rheumatoid Arthritis
Source: Mediators Inflamm. 2022 Oct 25;2022:4766992. doi: 10.1155/2022/4766992 (PMC9626244; doi:10.1155/2022/4766992)
Supplement: Supplementary Materials — See Table S1‑S5, Figures S1‑S4 in the Supplementary Material for comprehensive analysis. Table S1: basic characteristics of RA patients. Table S2: up- and down-regulated mRNA information of the top 10 differential peaks. Table S3: transcript information of the top 5 in the four-quadrant graph. Table S4: details of 36 transcripts with differential RNA methylation in PI3K-AKT signaling pathway. Table S5: mRNAs with differential m6A modification levels. Figure S1: the KEGG heatmap of upregulated mRNAs distribution information in RA synovium differentially expressed genes. Figure S2: the KEGG heatmap of down-regulated mRNAs distribution information in RA synovium differentially expressed genes. Figure S3: the KEGG heatmap of upregulated peaks in m6A modified apparent transcriptome. Figure S4: the KEGG heatmap down-regulated peaks in m6A modified apparent transcriptome. [file 4766992.f1.zip › Table S5 (1).docx]

Table S5 mRNAs with differential m6A modification levels

| Gene name | m6A log2FoldChange | m6A P-value | Gene log2FoldChange | Gene P-value | Change | Primer sequence | |
| --- | --- | --- | --- | --- | --- | --- | --- |
|  |  |  |  |  |  | Forward primer（5'→3'） | Reverse primer（5'→3'） |
| PTEN | -4.33380 | 0.01155 | -3.26925 | 0.01507 | down | ATGTTCAGTGGCGGAACTTG | GAACTTGTCTTCCCGTCGTG |
| ASPM | -4.29512 | 0.02018 | -2.60292 | 0.00001 | down | AGCCAGATTGGGTTTTGAGA | AGTTGTACACGGAGAGCAAA |
| SHCBP1 | -4.25813 | 0.00576 | -2.44337 | 0.00052 | down | AATTTGAAATGGCTGACGGG | TGCAGTCAGCTGGTTTATCA |
| ZBTB16 | 3.33756 | 0.00001 | 1.27367 | 0.00821 | up | CCCTCCTCGGCTCTCG | CAGGCATGAGGCTTTCTTTC |
| NXPH3 | 2.63251 | 0.00970 | 1.53510 | 0.01225 | up | AGGAAGATGCAACTGACTCG | CCACAGATGACCAGATAGAGG |
| SEMA4A | 2.18304 | 0.00006 | 2.03156 | 0.00199 | up | ACTGTGGCTGGTTCAAGG | CATAGTATCTGACCCTGGGC |
